# Transcriptomic Profiling of the Tumor Immune Microenvironment Reveals Prognostic Markers in mCRPC Patients Treated with LuPSMA Therapy

**DOI:** 10.7150/thno.113614

**Published:** 2025-08-30

**Authors:** Analena Handke, Leonor Lopes, Claudia Kesch, Christopher Darr, Elai Davicioni, Kuangyu Shi, Tugce Telli, Wolfgang P. Fendler, Ken Herrmann, Katharina Lückerath, Boris Hadaschik, Robert Seifert

**Affiliations:** 1 Department of Urology, University of Duisburg-Essen and German Cancer Consortium (DKTK), University Medicine Essen, Essen, Germany.; 2 Department of Urology, Ruhr-University Bochum, Marienhospital Herne, Herne, Germany.; 3 Department of Nuclear Medicine, University Hospital Bern, Inselspital, University of Bern, Bern, Switzerland.; 4 Veracyte, Inc, San Diego, California, USA.; 5 Department of Nuclear Medicine, University of Duisburg-Essen and German Cancer Consortium, (DKTK)-University Medicine Essen, Essen, Germany.

**Keywords:** prostate cancer, transcriptomic profiling, [^177^Lu]Lu-PSMA-617 therapy, Decipher prostate assay, PD-L2.

## Abstract

**Rationale:** The mode of action of [^177^Lu]Lu-PSMA-617 (LuPSMA) therapy is not fully understood and a relevant fraction of patients show treatment failure. Therefore, this study aimed to investigate the prognostic significance of immune suppression in the tumor immune microenvironment (TME) of LuPSMA therapy patients.

**Methods:** Tumor tissue samples from 61 patients, who were referred for LuPSMA from March 2018 until March 2022, were retrieved. Among these, 40 patients fulfilled all criteria and were therefore included in the analysis. Of these, 3 patients had two biopsies; prior and under systemic treatment, which is why we analyzed 43 samples: 29 (67%) with treatment-naïve tissues samples (cohort 1) and 14 (33%) during systemic treatment. Patients were followed up to assess overall survival. We examined gene expression and immune cell counts (derived from gene expression data) in the two sub-cohorts through transcriptome profiling with the Decipher prostate assay (Veracyte, San Diego, CA), a subset of these patients has been described previously.

**Results:** In the total cohort, the ratio of activated (M1)/naive (M0) macrophages (HR = 0.90 [0.84-0.98]; p = 0.009) was a significant prognosticator of OS. In cohort 1, PD-L2 expression (HR = 1.07 [1.02 - 1.11]; p = 0.003)) and the M1/M0 ratio signature (HR = 0.89 [0.81-0.99]; p = 0.026) were significant independent prognostic factors of OS when analyzed together in a multivariate analysis. AR gene expression was significantly elevated in cohort 2 compared to 1 (p < 0.001). Several DNA repair signatures analyzed were significantly higher in cohort 2 than in cohort 1 (p < 0.05). In cohort 2, PD-L2 expression (HR = 0.87 [0.77 - 0.98]; p = 0.017) emerged as an independent prognostic factor associated with improved OS when included in a multivariate model with the immune 190 score, a negative prognosticator in this analysis (HR = 1.25 [1.02 - 1.53]; p = 0.028).

**Conclusions:** The ratio of M1/M0 macrophages was associated with favorable outcome of LuPSMA in the total cohort of patients. In treatment-naive patient samples, *PD-L2* expression was associated with unfavorable, whereas M1/M0 macrophages with favorable outcomes, which might indicate that immune checkpoint inhibition could be a combination partner of LuPSMA therapy. In patient biopsy samples acquired after the start of systemic treatment, AR gene expression and DNA repair signatures appear to be significantly altered and *PD-L2* became a protective marker.

## Introduction

Metastatic castration resistant prostate cancer is incurable and presents challenges in optimal patient management. In recent years, prostate-specific membrane antigen (PSMA) targeted radioligand therapy with [^177^Lu]Lutetium PSMA-617 (LuPSMA) has emerged as a promising treatment option for patients with advanced prostate cancer 1. However, the observed variability in treatment responses among patients underlines the need for reliable additional biomarkers that can predict therapeutic outcomes and guide personalized treatment strategies.

The efficacy of LuPSMA therapy and ultimately patient outcome are influenced by a complex interplay of factors. In addition to PSMA-expression that co-determines tumor absorbed radiation dose, a currently studied element is the tumor microenvironment (TME), which encompasses the interactions between cancer cells and surrounding immune cells, amongst others 2-4. In various cancer entities other than prostate cancer, immune checkpoint molecules, particularly *programmed cell death protein 1* (*PD-1*) and its ligands, programmed death-ligand 1 and 2 (*PD-L1* and *PD-L2*), have been shown to play pivotal roles in tumor-mediated immune evasion mechanisms 5. In prostate cancer itself, only pembrolizumab was approved by the FDA for subtypes with microsatellite instability-high (MSI-H) 6. These molecules contribute to immune suppression in the TME and have emerged as important therapeutic as well as prognostic and predictive biomarkers for patient responses in various cancer entities 5.

Recent data suggest that combining PSMA targeting LuPSMA therapy and *PD-1/PD-L1* checkpoint inhibition can have a positive effect on targeting metastatic castration-resistant prostate cancer (mCRPC) 3,7. In addition, antigen presenting cells like dendritic cells or tumor-associated macrophages (TAMs) may influence the response to immunomodulatory therapy. For example, increased TAM infiltration, especially of the anti-inflammatory tumor-supportive M2 phenotype, correlates with poor prognosis in various cancers 8. Yet androgen deprivation therapy (ADT) and other androgen receptor pathway inhibitors (ARPI) can heavily affect the TME in prostate cancer and may render it difficult to prognosticate the outcome using only singular immune-related markers, which is why a comparison of individual transcriptomic markers between treatment naïve and pretreated patients is warranted.

A comprehensive analysis of the immunophenotype in mCRPC patients scheduled for treatment with LuPSMA is missing to date. Previously, we identified *PD-L2* and not *PD-L1* to be significantly prognostic in LuPSMA treated patients 9. However, the interplay with PD1, antigen presenting cells and tissue exposed to prior treatment is poorly understood. Therefore, the present study encompasses a broadened patient base (including that of our previous work 9) and an expanded analysis to investigate the prognostic significance of the immunophenotype in the TME of patients who receive LuPSMA therapy. To this end, both the presence of antigen presenting cells that were derived from transcriptomic data, as well as the levels of PD-1 signaling are studied. Both biopsy samples taken before and during/after systemic treatment are investigated, as this enables us to assess the influence of antiandrogen and other therapies on the outcome prognostication of LuPSMA.

## Methods

### Patient Selection

**Figure 1:**
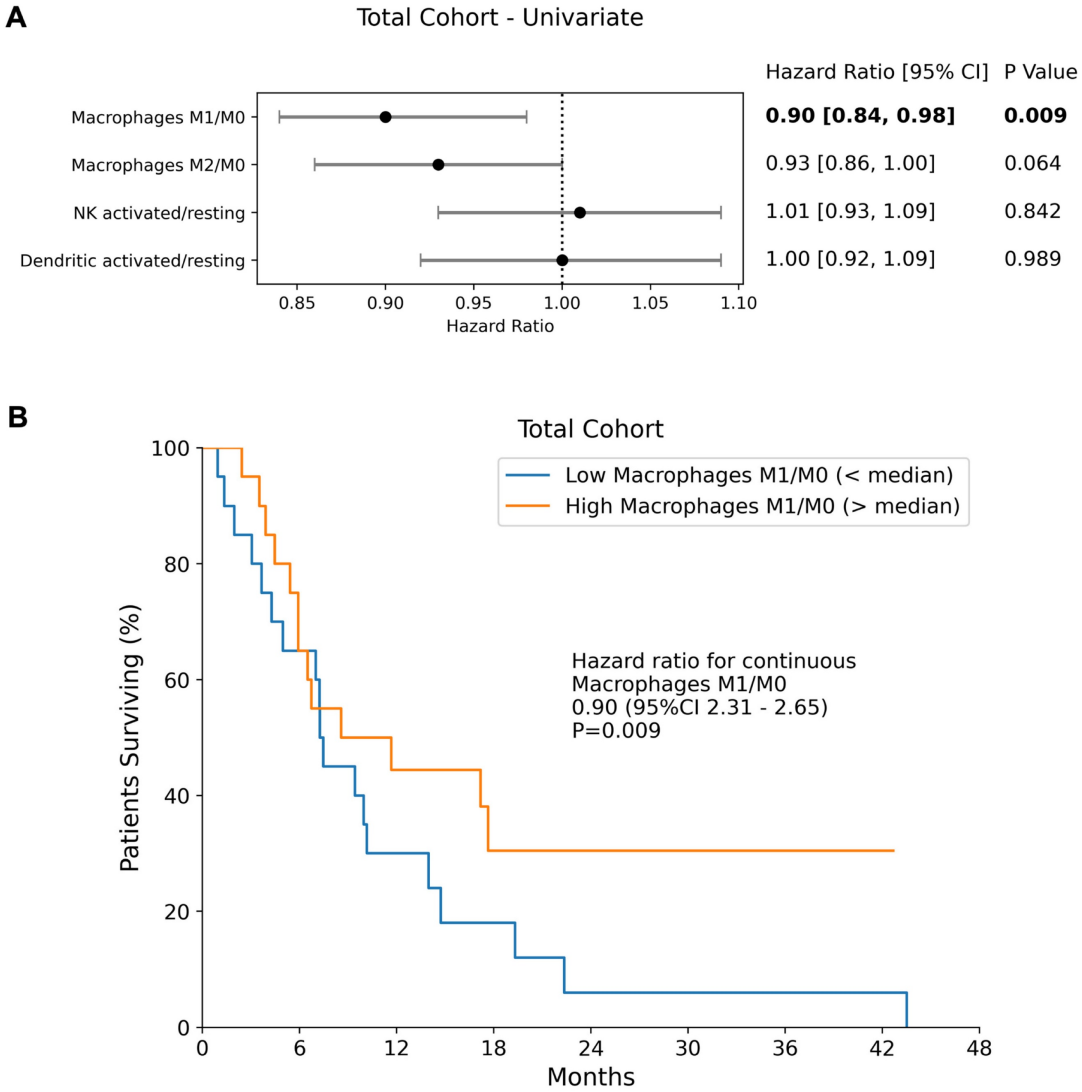
** Antigen Presenting Cells and Overall Survival. (A)** Forest plots of univariate Cox Proportional Hazards model in the Total Cohort for IEC ratios. Hazard ratios, 95% confidence intervals (95% CI) as well as p-values of each univariate cox model are presented.** (B)** The median overall survival of the total cohort is shown separately for patients with an M1/M0 ratio above and below the median.

We analyzed samples from 61 mCPRC patients who were referred for LuPSMA at the Nuclear Medicine Department of Essen University Hospital from March 2018 until March 2022 as part of routine clinical practice; all patients received LuPSMA treatment. Histopathologic formalin-fixed paraffin-embedded (FFPE) samples of primary tumor or metastases were acquired from the respective pathology institutes. After sample preparation and quality control, 40 patients were available for transcriptomic analyses, one patient had to be excluded since no further therapy was given after the bipsy. Of those, a subset of 23 patients has been reported on previously 9. The present cohort was divided into 29 patients who had treatment-naïve samples (cohort 1) and 14 patients from whom the biopsy samples were obtained during systemic treatment (cohort 2). Three patients had biopsies at both timepoints, which is why the total number of analyzed tissues is 43; for these patients, only the most recent tissue was chosen for analysis on the total cohort level. The time between biopsy and start of LuPSMA therapy was in mean 48 months (IQR 25;61) for cohort 1 and 8.5 months (IQR 4.5;21) for cohort 2, whereas 2 patients had their biopsy in mean 2 months (IQR 1.5;2.5) after the start of LuPSMA therapy. Patients were followed up to assess overall survival (OS) (Figure 1). OS was defined as the time from the initiation of LuPSMA therapy until death or loss to follow-up. In total, 3 patients had no PSA follow up. The study was conducted in accordance with the Declaration of Helsinki. The local ethics committee approved this retrospective study (Permission Number: 21-9882-BO) and waived the need for study specific consent.

### Transcriptomic Analysis

Material (FFPE) from either the diagnostic needle-core biopsy (minimum tumor tissue length of 0.5 mm) or from radical prostatectomy (≥ 0.5 mm²) was used, in both cases the highest-grade group was chosen for analysis using the Decipher prostate transcriptome assay (Veracyte, San Diego, CA), as described previously 9. Single Channel Array Normalization algorithm and other preprocessing steps were applied to the expression data to calculate relative expression genes as previously described 9,10. A compendium of locked gene expression signatures (n = 459) was retrieved from the Genomics Resource for Intelligent Discovery (GRID, version 3.0) database (Veracyte, San Diego, CA) 9. The analysis focused on DNA damage and repair ('core overall') and hallmarks of cancer interferon alpha response signatures as previously described 11,12. Among the retrieved tissue samples, 10 samples failed pathological review (insufficient tumor content remaining in the block), 1 sample failed cDNA amplification (RNA too degraded to amplify), 3 failed microarray quality control (gene expression profiles with low signal to noise) and 1 failed the analysis. In the end, transcriptomic analysis was available for 41 patients.

### Quantification of Tumor Immune Cell Count with Transcriptomic Data

The immune and stromal components within tumors were assessed using RNA expression data, employing techniques developed by Yoshihara and colleagues 13. This analysis was conducted using the "ESTIMATE" R package, version 2.0. We then employed the MySort tool to deconvolute the composition of tumor-infiltrating immune cells 14. To ensure positive values when applying proportions of tumor-infiltrating immune cells, we adjusted the immune content score by adding the absolute value of the minimum score in each cohort, plus one, to the derived immune score as described previously 15. These proportions were then multiplied by the estimated immune content to calculate the quantity of each cell type. This approach allowed for comparisons of immune cell quantities across samples within the same cohort.

### Further Analyses of Gene Expression and Transciptomic-Derived Immune Cell Count Data

We examined the influence of the TME, namely innate immune cells with antigen presenting capabilities activated/resting ratio (M1 and M2 macrophages, NK cells and dendritic cells; denoted in the following as innate effector cells (IECs)) and immune checkpoint inhibitors - *PD1, PD-L,PD-L2* and Cytotoxic T-Lymphocyte Associated Protein 4 (CTLA4) gene expression - as prognostic factors in the total cohort of patients selected for radioligand therapy. To further explore the influence of biopsy timing (before or after therapy start) we divided the total cohort into cohort 1 - patients that underwent biopsy before any treatment - and cohort 2 - patients that underwent biopsy under treatment. We further explored other immune factors that could be influencing *PD-L1/L2* prognostic value, namely immune 190 score as a broad marker of inflammatory activity and interferon type I gene expression as it is also stimulated by DNA-damage just as PD-L-activation. Finally, we explored differences in differentially expressed genes, transcriptomic-derived immune cell counts and DNA damage repair signatures between first and second sub-cohorts, because DNA damage is a simulator of PD-L-activation. All gene expression were normalized to ensure positive values, similarly to immune cells, and also scaled (multiplied by 100) for easier interpretation of hazard ratios.

### Statistical Analysis

Statistical analysis was performed in Python 3.9.12 with the libraries Lifelines 0.27.7 and Scipy 1.7.3. The assessment of variable skewness/normality was determined by the Shapiro-Wilk test and statistical tests or data transformations were performed accordingly. Descriptive data were presented as mean ± standard deviation for normally distributed parameters or median (interquartile range) for skewed parameters. Kaplan-Meier analysis was performed to estimate the median overall survival with 95% confidence interval (95% CI) and the survival curves of cohorts 1 and 2. Cox proportional hazards regression models were used to determine the significance of gene expression levels and transcriptomic-derived immune cells as predictors of overall survival (OS). Univariate Cox regression analyses were initially performed for each gene expression or transcriptomic-derived immune cells. To further assess the significance of certain markers, multivariate Cox regression analyses were conducted. All univariate and multivariate Cox models were corrected for patient age. Table S1 lists the skewed parameters; skewed parameters were log transformed for Cox regression. Hazard ratios (HR) and 95% CI as well as p-values are presented. Differences in gene expression and immune cell counts between first and second sub-cohorts were assessed by Mann-Whitney U test. Correlations were assessed by Spearman's test. Two-sided p-values < 0.05 were considered statistically significant.

## Results

### Patient Characteristics

Detailed patient characteristics for the total cohort are shown in Table 1, which shows all prior therapies before the start of LuPSMA.

### Analysis of the Total Cohort

Among the evaluated transcriptomic-derived IECs activation ratios, only the ratio of macrophages M1/M0 (HR = 0.90 [0.84 - 0.98]; p = 0.009) was a significant prognosticator of OS (Figure 1), with higher M1 suggesting better outcome. No other transcriptomic-derived immune cell ratio showed significant prognostic value in the full cohort (M2/M0; NK cells activated/resting, dendritic cells activated/resting). The expression levels of *PD-L2, PD-1*, *PD-L1* and CTLA4 were not found to be significant prognostic factors of overall survival in the combined cohort (p = 0.745, p = 0.273, p = 0.371 and p = 0.292 respectively).

**Figure 2:**
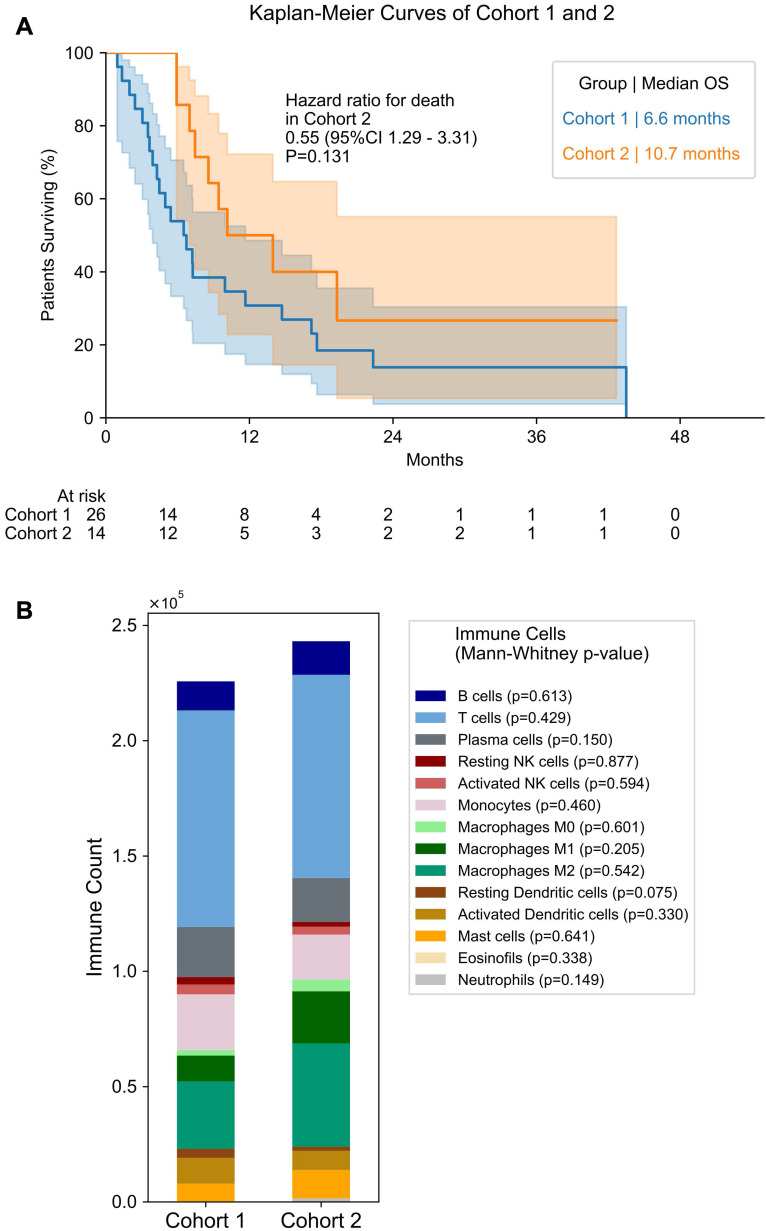
** Antigen Presenting Cells and Differentiation Between Cohort 1 and 2. (A)** Overall Survival for patients of cohort 1 and 2. Median overall survival is shown for both groups. Number of patients at risk in each time point is given below the plot. **(B)** Bar plot of immune cell count in cohort 1 and 2. Mann-Whitney test p value is shown for differences in each immune cell between cohorts.

As the overall cohort includes samples obtained before and during treatment, we separated the patients into 2 sub-cohorts: cohort 1 - patients that underwent biopsy before any treatment - and cohort 2 - patients that underwent biopsy under treatment. Table 2 provides an overview of the treatments received by the patients in cohort 2 up before the biopsy was taken. Median overall survival from start of LuPSMA was not significantly different between cohort 1 and 2 (6.6 months vs. 10.7 months, p = 0.131; Figure 2). The estimated 12-month overall survival rate was 26.9% (95% CI 11.9 - 44.5) for cohort 1 and 40.0% (95% CI 14.5 - 64.7) for cohort 2.

### Analysis of Cohort 1 (Biopsy Before Any Therapy)

#### Univariate Cox Proportional Hazards Model

For the subset of patients who underwent biopsy before any prostate-specific treatment, *PD-L2* (HR = 1.04 [1.01 - 1.08]; p = 0.018) and *PD-1* (HR = 1.09 [1.01 - 1.18]; p = 0.030) expression levels were significant prognostic factors of OS, while *PD-L1* (p = 0.567) and CTLA4 were not (p = 0.584) (partly with overlapping cohort already published as stated in the methods 9). In this cohort, no transcriptomic-derived IECs were significant prognosticators of OS in univariate analysis.

#### Multivariate Cox Proportional Hazards Model

**Figure 3:**
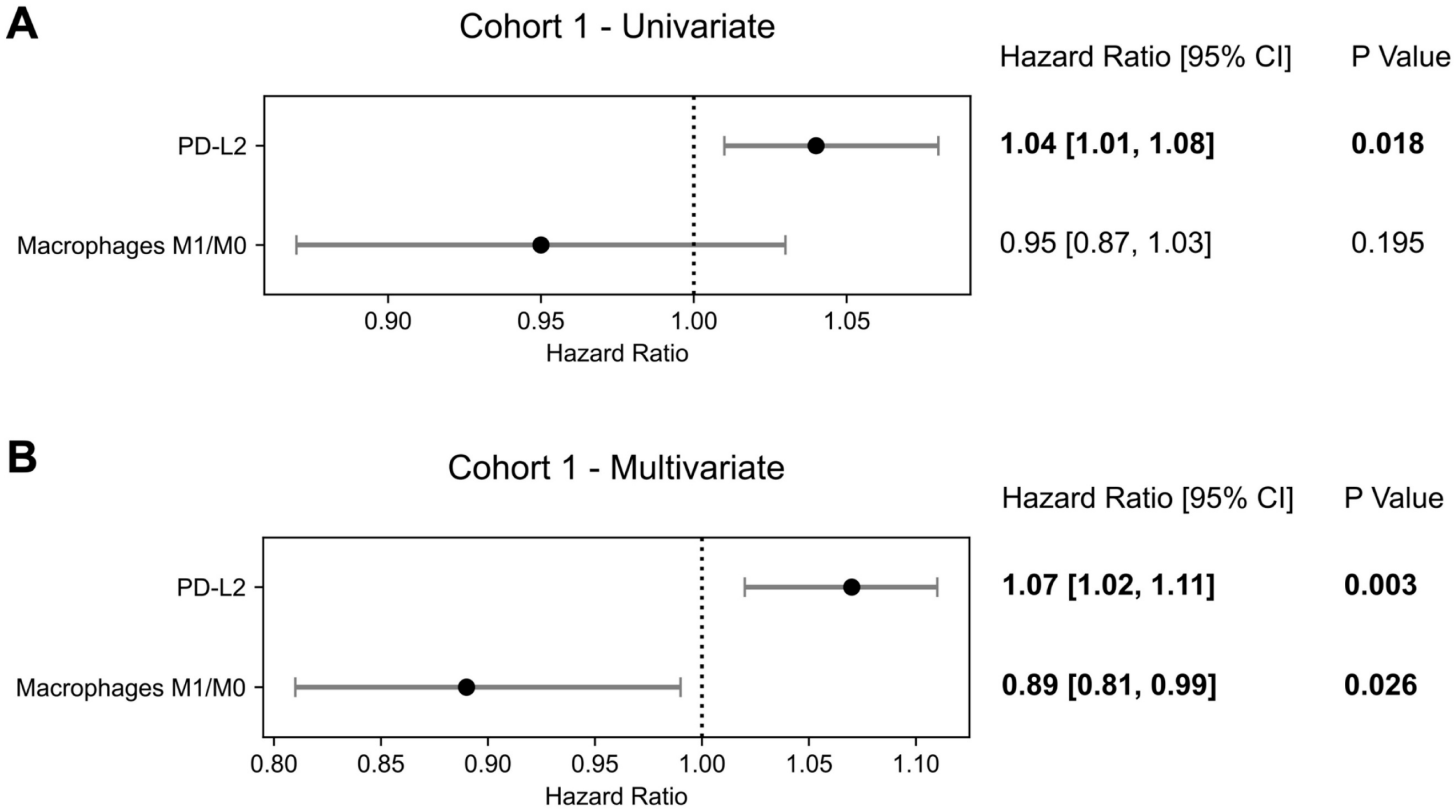
** Overall Survival Prognostication in Cohort 1.** Forest plots of Cox Proportional Hazards model for *PD-1, PD-L2* and macrophages M1/M0 signature in Cohort 1 in **(A)** a univariate analysis and **(B)** multivariate analysis. Hazard ratios, 95% confidence intervals (95% CI) as well as p-values of each univariate and for the multivariate cox model are presented.

**Figure 4:**
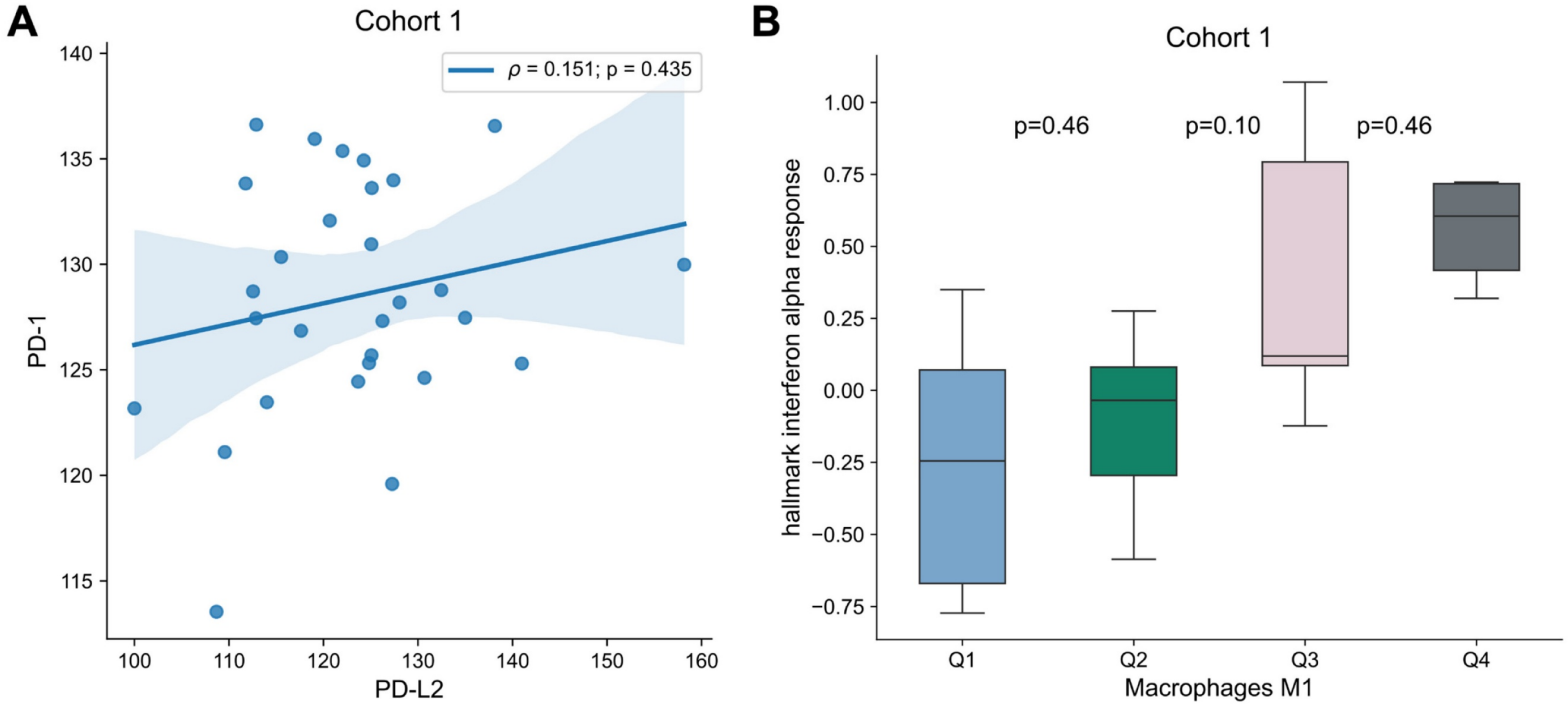
** Association of *PD-1* and *PD-L2* as well as M1 Differentiation. (A)** Correlation of *PD-1* and *PD-L2* expression for Cohort 1. Correlation was assessed by Spearman's test, and correlation coefficient and p-value are shown. **(B)** Box plots of “hallmark interferon alpha response” signature per macrophages M1 quartile. Differences were assessed by Mann-Whitney U test and p-values are shown.

To analyze the interplay between the targets for immune checkpoint inhibitors and the immune microenvironment, multivariable analysis with *PD-1, PD-L1*, *PD-L2 or CTLA4* and each transcriptomic-derived IEC ratio as covariates were performed (Table 3). When in a multivariate analysis with *PD-L2* expression, the ratio of macrophages M1/M0 (HR = 0.89 [0.81 - 0.99]; p = 0.026) became a significant prognostic factor of OS (Figure 3). No other transcriptomic-derived IEC ratio became significant after adjusting for *PD-1, PD-L1*, *PD-L2* or CTLA4 expression as covariates. Of notice, in a multivariate analysis with the transcriptomic-derived M1/M0 macrophage ratio and both *PD-L2* and *PD-1* as covariates, the latter was a significant protective factor (HR = 0.90 [0.81 - 0.99]; p = 0.026). Spearman's test showed no significant correlation (p = 0.435) between *PD-L2* and *PD-1* (Figure 4). Transcriptomic-derived M1 count was more pronounced in tissues with greater markers of response to interferon alpha expression (Figure 4).

### Analysis of Cohort 2 (Biopsy During Systemic Treatment)

#### Univariate Cox Proportional Hazards Model

In the cohort of patients who underwent biopsy after treatment begin, none of the evaluated markers (transcriptomic-derived IECs counts/ratios, *PD-L2, PD-1, PD-L1, CTLA4*) were significant prognostic factors of OS in the univariate Cox regression analysis.

#### Multivariate Cox Proportional Hazards Model

To explore specific immune factors that could be contributing to *PD-1, PD-L1*, *PD-L2 and CTLA4* influence on survival, multivariate analyses with *PD-1, PD-L1, PD-L2 and CTLA4* and each transcriptomic-derived IEC count/ratio as covariates were performed. Hazard ratios, 95% confidence intervals and p-values are shown in Table 4. In none of these models, *PD-L2* became significantly associated with OS, nor were any of the transcriptomic-derived IEC ratios.

**Figure 5:**
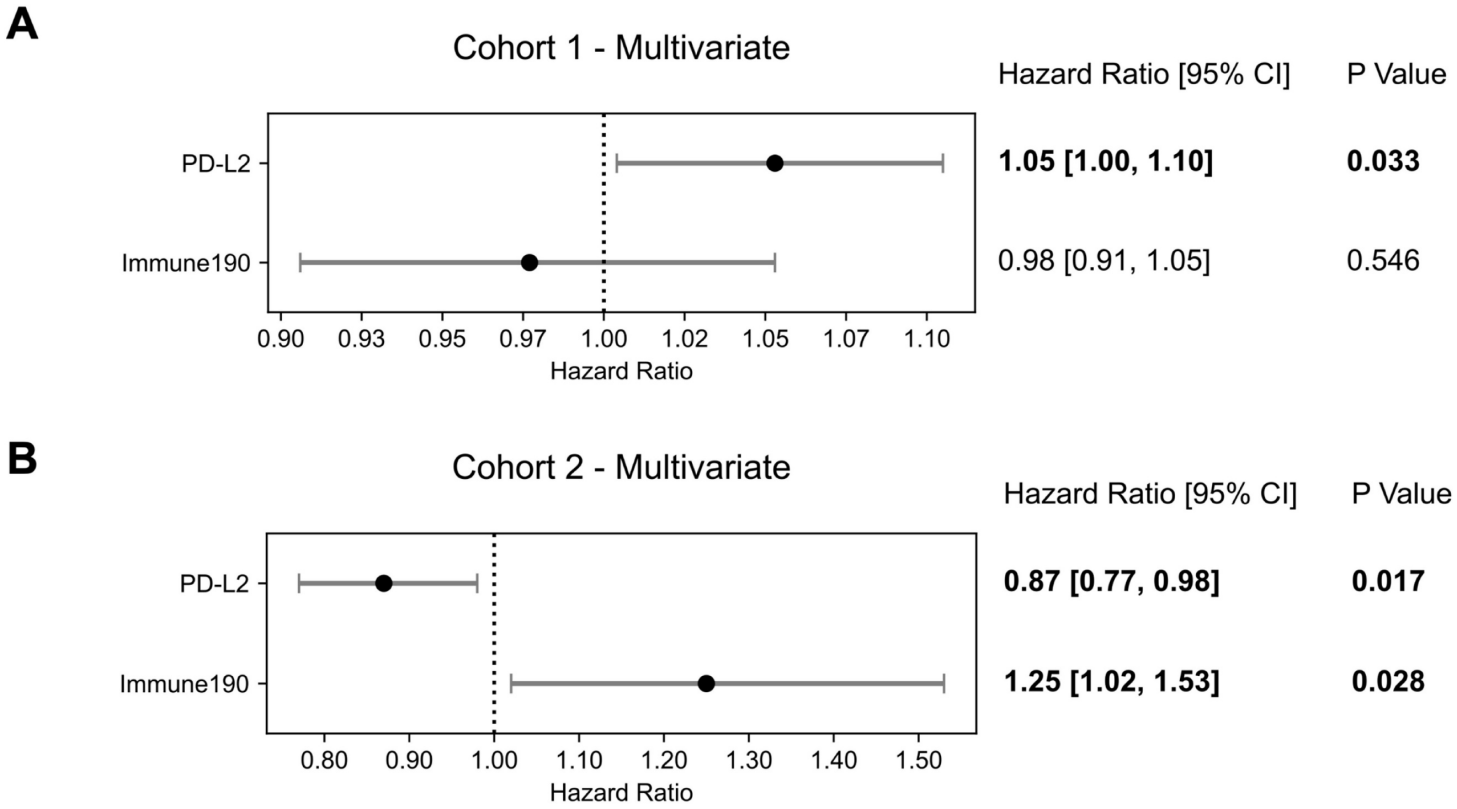
** Multivariate Overall Survival Prognostication Including Immune Activation.** Forest plots of multivariate Cox Proportional Hazards model with *PD-L2* and immune190 signature as covariates for **(A)** cohort 1 and **(B)** cohort 2. Hazard ratios, 95% confidence intervals (95% CI) as well as p-values are presented**.**

We hypothesized that the immunophenotype was altered by the influence of the systemic treatment and therefore searched for impact of other immune factors; as an initial naïve attempt we performed adjusted multivariate analysis with *PD-L2* and immune190 signature as covariates, which is a broad inflammatory signature included in GRID 16. Both immune190 (HR = 1.25 [1.02 - 1.38]; p = 0.028) and *PD-L2* were significantly associated with OS (HR = 0.87 [0.77 - 0.98]; p = 0.017; Figure 5).

Given the significant prognostication with the tumor inflammation surrogate marker immune190, specific interferons were investigated as covariates (Table S2). This identified IFNA6 (HR = 0.92 [0.85 - 0.99]; p = 0.039) together with *PD-L2* (HR = 0.97 [0.93 - 0.99]; p = 0.036) as significant prognosticators; interestingly, *PD-L2* and *IFNA6* were protective.

### Differences Between Cohort 1 and 2

**Figure 6:**
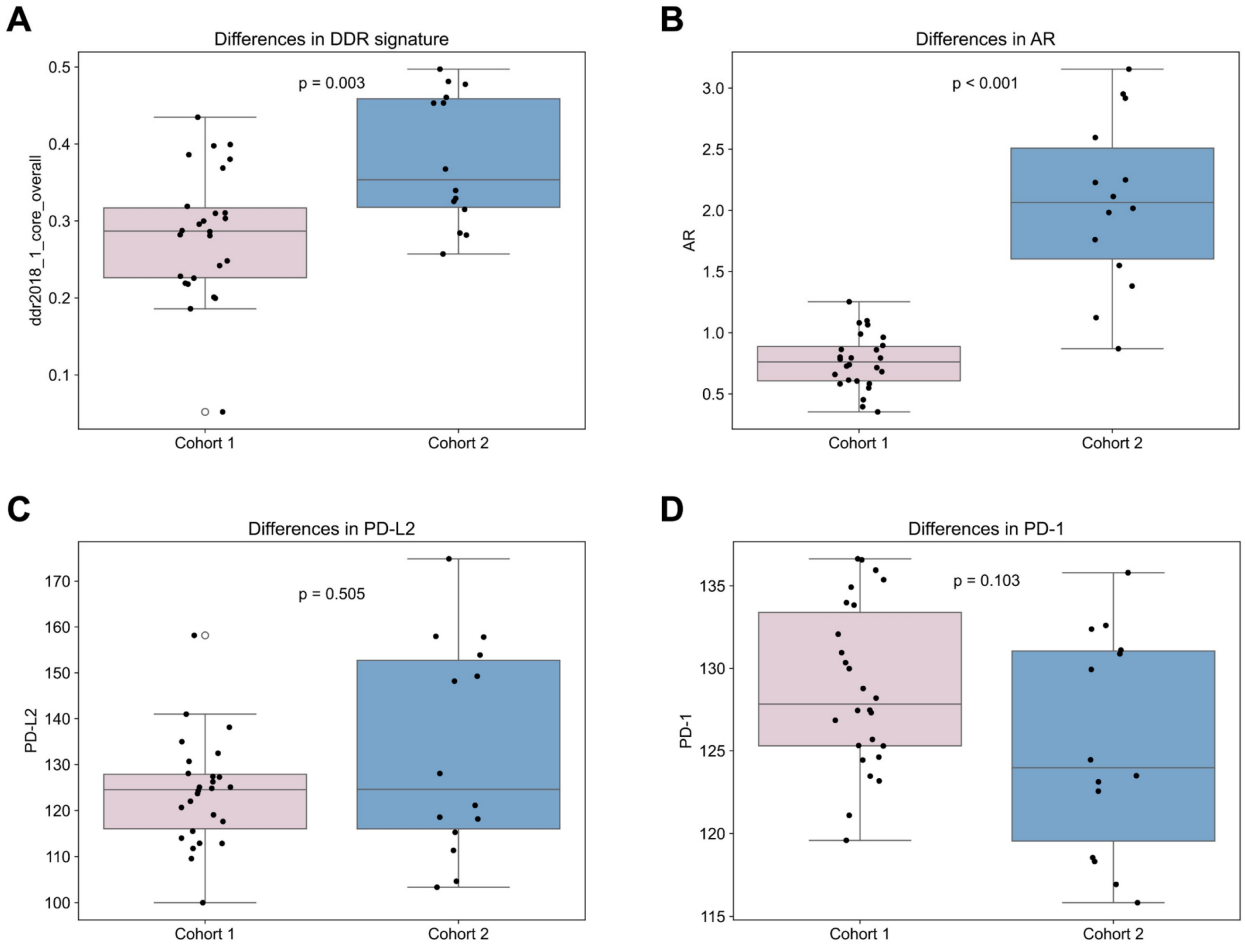
** Comparison of Cohort 1 and 2.** Differences between cohort 1 and 2 in **(A)** DNA damage response signature (signature “DDR core signature”^11^); **(B)** androgen receptor (AR) gene; **(C)** *PD-L2* and **(D)** *PD-1*.

Comparing treatment-naïve samples to samples obtained after therapy begin, the androgen receptor (AR) gene expression was significantly elevated in cohort 2 compared to 1 (p < 0.001; Figure 6). In addition, many DNA repair signatures analyzed were significantly higher in the cohort 2 than in cohort 1, namely ddr2018_1_core_overall (p = 0.003; Figure 6), ddr2018_1_core_base_excision_repair (p = 0.002), ddr2018_1_core_damage_sensor (p = 0.015), ddr2018_1_core_homologous_recomination (p = 0.001), ddr2018_1_core_mismatch_repair (p = 0.035) ddr2018_1_core_nonhomologous_end_ joining (p = 0.004) and ddr2018_1_core_nucleotide_excision_repair (p = 0.030). No significant differences were found between cohorts in *PD-1, PD-L1* or *PD-L2* expression (Figure 6). Also, no significant differences were found in transcriptomic derived overall immune cell content between the first and second cohort (Figure 2).

## Discussion

Building on our previous pilot study, the tumor immune microenvironment of patients with prostate cancer who received LuPSMA therapy was examined 9. We limited the immune cell analysis to innate cells with antigen presenting capabilities and discovered that the ratio of M1 polarized over resting macrophages (M1/M0) in prostate cancer tissue is a significant prognosticator of overall survival. Patients in whom the biopsy was acquired after the start of systemic therapy comprising ADT had significantly elevated DNA damage repair signature and androgen receptor levels. Interestingly, in those patients, *PD-L2* alone was not prognostic for outcome, but became relevant when adjusted for immune190 or specifically type 1 interferon (A6). This indicates a potential role of immune evasion in patients who are treated with radioligand therapy and warrants further trials to corroborate the preliminary findings.

As LuPSMA therapy is a theranostic treatment option, PSMA-PET is used to select ideal therapy candidates 17. Amongst other parameters, the average PSMA uptake of all metastases, the total tumor volume and PSMA positivity have been identified as prognostic factors of patient outcome 4,18,19. In routine clinical practice, PSMA expression of the tumor burden is assessed and patients with low target expression are excluded. However, still a considerable fraction of patients is not sufficiently responding to LuPSMA therapy, which is why molecular markers like circulating tumor DNA (ctDNA) have garnered more attention. Inter alia, those analyses found specific receptor amplifications to be associated with poor response in patients treated with LuPSMA alone, specific ctDNA alterations to be associated with poorer outcome of patients treated with Lu-PSMA and idronoxil, and ctDNA levels to be associated with better outcome when treated with LuPSMA than with ARPI 20-23. However, those approaches generally are not able to capture the tumor immune microenvironment compared to transcriptomic analysis of tumor tissue.

Growing evidence suggest that radiation can increase the immune responses to prostate cancer 24. This contrasts with other anticancer therapies like docetaxel, which failed to demonstrate an improvement of response when combined with checkpoint inhibition 25. We hypothesized that innate immune cells with antigen presenting capabilities are the first effector of immune stimulation, which is why we limited the preliminary analysis to this cell type 26. Indeed, the fraction of M1 differentiated macrophages over resting macrophages in the tumor as inferred by gene expression signatures was a significant protective factor for overall survival. This subpopulation of M1 macrophages is proinflammatory and might therefore promote anticancer immune response, which is in line with previous research outside LuPSMA therapy 27. Still, this is controversial, as macrophage infiltration in general is seen as bad prognosticator in prostate cancer, which highlights the preliminary nature of the evidence 28.

The present cohort is heterogeneous regarding the sampling of the tumor tissue, some patients were treatment naïve with tissues collected quite long before start of LuPSMA, whereas other underwent ADT and other systemic therapy lines prior to tissue sampling. The treatment naïve cohort had significantly lower levels of DNA damage repair pathway activations and lower levels of androgen receptor gene expression. These findings suggest potential treatment-associated changes in DNA repair activity. However, DNA repair signatures were not included in survival analyses, and their prognostic significance remains exploratory. Previous findings showed reduced efficacy of DNA repair in patients treated with ADT, which could cause accumulation of DNA damage and in turn higher activation of DNA repair pathways 29.

Previously, we showed that *PD-L2* gene expression is a significant prognosticator of outcome in patients treated with LuPSMA therapy 9. The cohort of the present manuscript comprises patients from the same cohort, but *PD-L2* was not prognostic in the expanded total cohort, which is related to the inclusion of many non-treatment naïve biopsies here. In the cohort of treatment naïve biopsies, *PD-L2* gene expression was associated with worse outcomes, whereas M1/M0 fraction of activated macrophages are protective and associated with better outcomes. This is in line with a recent report on the potentially beneficial effect of checkpoint inhibition therapy in a cohort of patients who received LuPSMA therapy 3. Also, this has been indicated by preclinical experiments 30. In addition, the preliminary findings suggests that *PD-L2* and *PD-1* could contribute to an axis that mediates the immune escape and that M1 macrophages mediate immune activating effects. In cohort 2 (receiving systemic treatment before biopsy), *PD-L2* alone or in combination with the transcriptomic-derived M1 macrophages count was not prognostic; however, DNA damage repair genes were higher in this cohort, which indicated greater degree of DNA damage and damage repair capacity. DNA damage can activate both immune escape via a STAT1/3 mediated pathway that upregulates *PD L2* expression and inflammation via a NF-κB pathway type 1 interferon (Figure S1) 5,25. Both effects are partially conflicting, as the immune stimulatory effect of interferon secretion could be antagonized by increased immune suppression via *PD-L2*, amongst others. Therefore, we hypothesized that pro- and anti-inflammatory markers needed to be jointly regarded and thus we investigated the role of immune-stimulatory markers together with *PD-L2* in cohort 2.

After accounting for this effect, *PD-L2* and the aggregate immune infiltration signature Immune190 were significantly associated with the outcome in cohort 2. Interestingly, *PD-L2* is protective in cohort 2, contrasting with the negative effect in cohort 1. This discrepant finding may be partly explained by the induction of DNA damage through systemic therapies, which leads to *PD-L2* and NF-κB pathway activation (Figure S1). Thus, *PD-L2* activation may have a dual role: as negative prognosticator because of the induced immune escape, but also as positive prognostic indicator of treatment efficacy. This suggests that the immune microenvironment of prostate cancer is significantly changed by the start of systemic treatments.

Tumor inflammation measured by a transcriptomic signature for aggregate immune infiltration in cohort 2 is negatively associated with the outcome 16. Previous reports show that chronic tumor inflammation, in particular persistent activation of interferon type 1 signaling pathways, can lead to T-cell exhaustion and that T-cell exhaustion as well as inflammatory markers can promote tumor progression in prostate cancer 31-33. This could also explain the paradoxical protective effect of *PD-L2* levels in cohort 2. In this context, *PD-L2* may also have a protective effect as it can modulate excessive immune activation, preventing immune exhaustion and enabling better cancer control.

The current study has several limitations. First, it includes samples from patients that have been analyzed in a prior study (9) testing multiple hypotheses. Here, the cohort was significantly enlarged, and new parameters were extracted, exemplarily including immune cell composition, interferon and *PD1* expression. In addition, this study distinguished for the first time between the time points of sample collection, with cohort 1 taking place before therapy and cohort 2 during systemic therapy. The analysis was conducted retrospectively and includes a relatively small number of patients. Future prospective studies incorporating both transcriptomic and immunohistochemistry or immunofluorescence methods, will be important to validate and expand upon these observations. Given the limited sample size, we were only able to assess a subset of variables in multivariate models. As a result, we cannot fully determine the extent to which each factor is independent of other potential prognostic markers. Nevertheless, the findings offer relevant directions for further confirmatory studies in the emerging landscape of radioligand therapy for prostate cancer and the growing number of trials that are in the recruitment and preparation phase for concomitant immunomodulatory therapy.

## Conclusion

Analysis of the innate immune cell count with antigen presenting capabilities derived from transcriptomic analysis and the expression of markers for immune escape is feasible in patients undergoing LuPSMA therapy. In samples acquired in treatment-naive patients, PD-L2 is a significant prognosticator of OS, as shown previously in a partly overlapping cohort. In patients who underwent tumor biopsy after the start of systemic treatment, PD-L2 was only an independent prognostic factor when adjusted for tumor inflammation in a multivariate analysis. The ratio of M1/M0 activated macrophages is a significant prognosticator of OS in the entire cohort. The TME of prostate cancer seems to be significantly changed after start of systemic treatment, which makes the identification of prognostic biomarkers challenging. Still, the results suggest that checkpoint inhibition may increase the efficacy of LuPSMA therapy, but further studies are needed to understand their potentially protective role to limit tumor inflammation.

## Supplementary Material

Supplementary figures and tables.

## Figures and Tables

**Table 1:** Patient Characteristics

Patient characteristics	Total cohort (n = 40)	Treatment-naïve tissue sample Cohort 1 (n = 29)	During therapy tissue sample Cohort 2 (n = 14)
Age [years]	68 [63-76]	67 [63-76]	68 [64-72]
Median overall Survival [months]	8 [5-15]	6.7 [3.9-17.2]	10.8 [7.8-14.5]
Previous therapies before LuPSMA			
*Docetaxel*	34 (85%)	25 (86%)	12 (86%)
*Cabazitaxel*	13 (33%)	9 (31%)	4 (29%)
*ARPI*	37 (93%)	27 (93%)	14 (100%)
*Radium-223*	6 (15%)	4 (14%)	2 (14%)
PSMA therapy			
*Median total number of cycles*	3 [2-4]	2 [1-4]	4 [3-4]
*Median total activity [GBq]*	24.2 [12.4-28.2]	17.8. [11.7-24.8]	26.5 [24.3-34]
*Average activity per cycle [GBq]*	6.4 [6.2-6.5]	6.2 [6.2-6.4]	6.6 [6.5-6.8]
Baseline blood parameters			
*Hemoglobin [g/dl]*	10.8 [9.4-12.1]	10.2 [9.2-9.9]	11.8 [10.3-12]
*Thrombocytes/nl*	241 [201-319]	231 [197-284]	317 [274-354]
*PSA [ng/ml]*	107 [51-362]	190 [79-568]	56 [25-133]
*LDH [U/l]*	334 [231-578]	384 [232-599]	298 [234-445]

95% confidence interval is given in parenthesis, the interquartile range [Q1-Q3] in brackets. Unless otherwise stated, the data reflect the median and the interquartile range.

**Table 2:** Systemic Therapies Before Biopsy in Cohort 2

Patient characteristics	Quantity (n = 14)	Duration in months [m] resp. cycles [c] (n = 14)
ARPI	13 (93%)	
*Abiraterone*	10 (71%)	16.5 [6.5-21] [m]
*Enzalutamide*	11 (79%)	6 [5-9.5] [m]
*Apalutamide*	0	
Chemotherapy	10 (71%)	
*Docetaxel*	10 (71%)	4.4 [3.3-5.1] [c]
*Cabazitaxel*	3 (20%)	4 [3-3.4] [c]

95% confidence interval is given in parenthesis, the interquartile range [Q1-Q3] in brackets. Unless otherwise stated, the data reflect the median and the interquartile range.

**Table 3:** Identification of Relevant IMC-Subtype for Outcome Prognostication in Cohort 1

	*PD-1*		*PD-L1*		*PD-L2*		*CTLA4*	
	HR [95%CI]	p-value	HR [95%CI]	p-value	HR [95%CI]	p-value	HR [95%CI]	p-value
*PD-1/PD-L1/ PD-L2/CTLA4*	1.10 [1.01 - 1.18]	**0.022**	6.20 [0.02 - 2602.13]	0.554	1.07 [1.02 - 1.11]	**0.003**	4.37 [0.01 - 3679.60]	0.668
**Macrophages M1/M0**	0.93 [0.86 - 1.02]	0.122	0.95 [0.87 - 1.03]	0.192	0.89 [0.81 - 0.99]	**0.026**	0.93 [0.83 - 1.05]	0.219
*PD-1/PD-L1/ PD-L2/CTLA4*	1.09 [1.01 - 1.17]	**0.020**	5.90 [0.02 - 2112.84]	0.554	1.06 [1.02 - 1.10]	**0.006**	1.40 [0.01 - 410.57]	0.907
**Macrophages M2/M0**	0.93 [0.85 - 1.02]	0.119	0.95 [0.87 - 1.03]	0.205	0.91 [0.83 - 1.00]	0.057	0.94 [0.85 - 1.05]	0.264
*PD-1/PD-L1/ PD-L2/CTLA4*	1.08 [0.99 - 1.18]	0.079	7.05 [0.02 - 2418.30]	0.512	1.04 [1.01 - 1.08]	**0.022**	0.29 [0.00 - 29.65]	0.600
**NK cells activated/resting**	1.025 [0.93 - 1.14]	0.635	1.07 [0.98 - 1.18]	0.142	1.07 [0.97 - 1.17]	0.182	1.07 [0.98 - 1.18]	0.153
*PD-1/PD-L1/ PD-L2/CTLA4*	1.09 [1.01 - 1.18]	**0.033**	4.18 [0.01 - 2648.81]	0.664	1.04 [1.01 - 1.08]	**0.017**	0.34 [0.00 - 42.53]	0.661
**Dendritic cells activate/resting**	1.02 [0.92 - 1.12]	0.720	1.03 [0.93 - 1.13]	0.597	1.03 [0.94 - 1.14]	0.512	1.03 [0.93 - 1.13]	0.580

Hazard ratios, 95% confidence intervals (95% CI) as well as p-values of each IEC counts/ratios is presented for each multivariate model (with either *PD-1, PD-L1*, *PD-L2 or CTLA4*).

**Table 4:** Identification of Relevant IMC-Subtype for Outcome Prognostication in Cohort 2

	PD-1		PD-L1		PD-L2		CTLA4	
	HR [95%CI]	p-value	HR [95%CI]	p-value	HR [95%CI]	p-value	HR [95%CI]	p-value
PD-1/PD-L1/ PD-L2	0.94 [0.83 - 1.06]	0.315	0.01 [0.00 - 54.12]	0.304	0.98 [0.95 - 1.02]	0.406	7.11 [0.00 - 14677.44]	0.615
**Macrophages M1/M0**	0.92 [0.79 - 1.07]	0.256	0.97 [0.83 - 1.14]	0.724	0.97 [0.82 - 1.14]	0.711	0.91 [0.75 - 1.09]	0.294
PD-1/PD-L1/ PD-L2	0.95 [0.84 - 1.067]	0.376	0.00 [0.00 - 51.78]	0.247	0.98 [0.95 - 1.02]	0.277	1.54 [0.00 - 1979.91]	0.906
**Macrophages M2/M0**	0.96 [0.83 - 1.11]	0.550	1.02 [0.87 - 1.19]	0.848	0.99 [0.87 - 1.14]	0.932	0.96 [0.82 - 1.12]	0.604
PD-1/PD-L1/ PD-L2	0.96 [0.85 - 1.09]	0.496	0.01 [0.00 - 15.77]	0.207	0.99 [0.95 - 1.03]	0.532	5.49 [0.01 - 3665.90]	0.608
**NK cells activated/resting**	0.91 [0.78 - 1.05]	0.202	0.90 [0.76 - 1.06]	0.194	0.92 [0.78 - 1.10]	0.356	0.88 [0.75 - 1.04]	0.146
PD-1/PD-L1/ PD-L2	0.95 [0.84 - 1.07]	0.394	0.01 [0.00 - 17.47]	0.202	0.98 [0.95 - 1.02]	0.323	1.37 [0.00 - 907.96]	0.925
**Dendritic cells activate/resting**	1.00 [0.84 - 1.19]	0.998	1.00 [1.00 - 1.00]	0.411	1.00 [1.00 - 1.00]	0.600	1.00 [1.00 - 1.00]	0.393

Hazard ratios, 95% confidence intervals (95% CI) as well as p-values of each IEC counts/ratios is presented for each multivariate model (with either *PD-1, PD-L1*, *PD-L2 or CTLA4*)
