# Supplementary material for: Transcriptomic Profiling of the Tumor Immune Microenvironment Reveals Prognostic Markers in mCRPC Patients Treated with LuPSMA Therapy
Source: Theranostics. 2025 Aug 30;15(18):9447–58. doi: 10.7150/thno.113614 (PMC12486015; doi:10.7150/thno.113614)

Supplementary Materials

Table S1 – List of Skewed Parameters

Skewed parameters were log-transformed before Cox regression. Shapiro-Wilk was used.

Total Cohort	Cohort 1	Cohort 2
PD-L1	PD-L1	PD-L1
PD-L2		
Macrophages M1/M0	Macrophages M1/M0	Macrophages M1/M0
Macrophages M2/M0	Macrophages M2/M0	Macrophages M2/M0
Dendritic cells activated/resting	Dendritic cells activated/resting	
NK cells activated/resting	NK cells activated/resting	NK cells activated/resting

Table S2 – Multivariate Cox Regression of PD-L2 Expression with all Interferon Type 1 Genes

	HR [95%CI]	*p-value*
PD-L2	0.98 [0.94 - 1.01]	0.230
IFNA1	1.02 [0.94 - 1.11]	0.567
PD-L2	0.97 [0.93 - 1.01]	0.184
IFNA2	0.91 [0.76 - 1.10]	0.339
PD-L2	0.99 [0.95 - 1.02]	0.417
IFNA4	1.04 [0.95 - 1.15]	0.387
PD-L2	0.98 [0.95 - 1.01]	0.253
IFNA5	0.91 [0.80 - 1.03]	0.126
PD-L2	0.97 [0.93 – 1.00]	0.036
IFNA6	0.92 [0.85 -1.00]	0.039
PD-L2	0.98 [0.94 - 1.02]	0.319
IFNA7	1.01 [0.90 - 1.13]	0.883
PD-L2	0.98 [0.94 - 1.02]	0.229
IFNA8	0.93 [0.80 - 1.07]	0.280
PD-L2	0.98 [0.95 - 1.02]	0.334
IFNA10	1.03 [0.92 - 1.14]	0.648
PD-L2	0.99 [0.95 - 1.02]	0.363
IFNA13	0.90 [0.78 - 1.03]	0.128
PD-L2	0.98 [0.94 - 1.01]	0.221
IFNA14	0.95 [0.86 - 1.04]	0.273
PD-L2	0.98 [0.95 - 1.01]	0.244
IFNA16	1.00 [0.94 - 1.06]	0.986
PD-L2	0.98 [0.95 - 1.02]	0.381
IFNA17	1.04 [0.94 - 1.17]	0.445
PD-L2	0.97 [0.94 - 1.01]	0.149
IFNA21	0.93 [0.79 - 1.09]	0.353
PD-L2	0.98 [0.95 - 1.01]	0.225
IFNB1	0.97 [0.87 - 1.09]	0.616
PD-L2	0.96 [0.91 - 1.01]	0.123
IFNE	0.83 [0.68 - 1.02]	0.072
PD-L2	0.98 [0.94 - 1.02]	0.233
IFNW1	0.97 [0.80 - 1.18]	0.765
PD-L2	0.97 [0.93 - 1.01]	0.162
IFNK	1.01 [0.98 - 1.04]	0.419

Figure S1 – Simplified Tumor Microenvironment

DNA damage induces the activation of two opposing pathways: one immunostimulatory pathway leads to increased release of interferon alpha via the activation of NF-κB and type 1 interferon, among others, and thus to activation of the antigen-presenting cells. The second pathway contributes to increased immune evasion via increased reading of *PD-L1/2*. Interferon gamma leads to increased immune evasion in the tumor cell itself. On the other hand, it also leads to an activation of the antigen-presenting cells by stimulating the differentiation of macrophages. Figure based on Shi et al. [5] Created with Biorender.

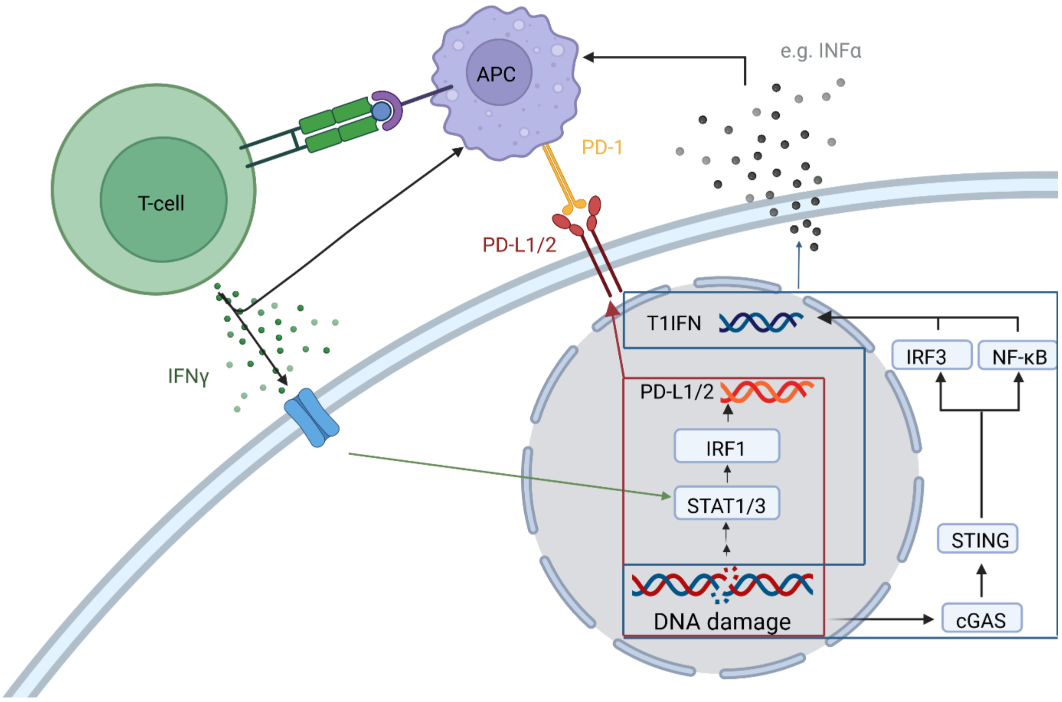

Supplement: Supplementary file 1 — Supplementary figures and tables. [file thnov15p9447s1.pdf]
